# Utilizing Organoid and Air-Liquid Interface Models as a Screening Method in the Development of New Host Defense Peptides

**DOI:** 10.3389/fcimb.2020.00228

**Published:** 2020-05-20

**Authors:** Ka-Yee Grace Choi, Bing Catherine Wu, Amy Huei-Yi Lee, Beverlie Baquir, Robert E. W. Hancock

**Affiliations:** ^1^Department of Microbiology and Immunology, Centre for Microbial Diseases and Immunity Research, University of British Columbia, Vancouver, BC, Canada; ^2^Department of Molecular Biology and Biochemistry, Simon Fraser University, Burnaby, BC, Canada

**Keywords:** host-defense peptide, organoid, air-liquid interface, disease model, drug screening, antimicrobial resistant organisms

## Abstract

Host defense peptides (HDPs), also known as antimicrobial peptides, are naturally occurring polypeptides (~12–50 residues) composed of cationic and hydrophobic amino acids that adopt an amphipathic conformation upon folding usually after contact with membranes. HDPs have a variety of biological activities including immunomodulatory, anti-inflammatory, anti-bacterial, and anti-biofilm functions. Although HDPs have the potential to address the global threat of antibiotic resistance and to treat immune and inflammatory disorders, they have yet to achieve this promise. Indeed, there are several challenges associated with bringing peptide-based drug candidates from the lab bench to clinical practice, including identifying appropriate indications, stability, toxicity, and cost. These challenges can be addressed in part by the development of innate defense regulator (IDR) peptides and peptidomimetics, which are synthetic derivatives of HDPs with similar or better efficacy, increased stability, and reduced toxicity and cost of the original HDP. However, one of the largest gaps between basic research and clinical application is the validity and translatability of conventional model systems, such as cell lines and animal models, for screening HDPs and their derivatives as potential drug therapies. Indeed, such translation has often relied on animal models, which have only limited validity. Here we discuss the recent development of human organoids for disease modeling and drug screening, assisted by the use of *omics* analyses. Organoids, developed from primary cells, cell lines, or human pluripotent stem cells, are three-dimensional, self-organizing structures that closely resemble their corresponding *in vivo* organs with regards to immune responses, tissue organization, and physiological properties; thus, organoids represent a reliable method for studying efficacy, formulation, toxicity and to some extent drug stability and pharmacodynamics. The use of patient-derived organoids enables the study of patient-specific efficacy, toxicogenomics and drug response predictions. We outline how organoids and *omics* data analysis can be leveraged to aid in the clinical translation of IDR peptides.

## Introduction

The development of antibiotics is one of the greatest advances in modern medicine. However, excessive and improper uses of antibiotics have led to the rapid increase in antimicrobial resistant (AMR) organisms. Given the dearth of new antibiotics, AMR infections have become one of the most serious global health issues. AMR organisms are associated with high morbidity and mortality, and contribute to high economic burden (Zhen et al., 2019). According to the Centers for Disease Control and Prevention (CDC), at least 2.8 million Americans are infected by AMR organisms annually, with 35,000 people succumb to the infection (www.cdc.gov/drugresistance/index.html), although this situation may in fact be far worse since ~210,000 die of sepsis annually due to the failure of the front line therapy, antibiotics. In Canada, the Council of Canadian Academies (CCA) reported that AMR organisms mediated infection have cost the health care system >$2 billion, and claimed 5,400 Canadian lives in 2018 (cca-reports.ca/antimicrobial-resistance-poses-significant-risk-to-people-the-economy/). The urgent need for the development of new therapies against AMR organisms, was indicated by the World Health Organization (WHO) publication of a global action plan containing a global priority list of 12 families of AMR organisms that pose the greatest threat to human health, to guide research and development of new antibiotics (World Health Organization, 2015, 2017). The CDC has also stated that “without urgent action now, we are on the verge of returning to a pre-antibiotic era, where common infections and injury can become fatal, and surgeries and chemotherapy will become impossible” (www.cidrap.umn.edu/news-perspective/2016/10/cdc-chief-antibiotic-resistance-scary-threat-modern-medicine). It is thus evident that there is an urgent need to develop new strategies to fight against AMR organisms.

**Figure 1:**
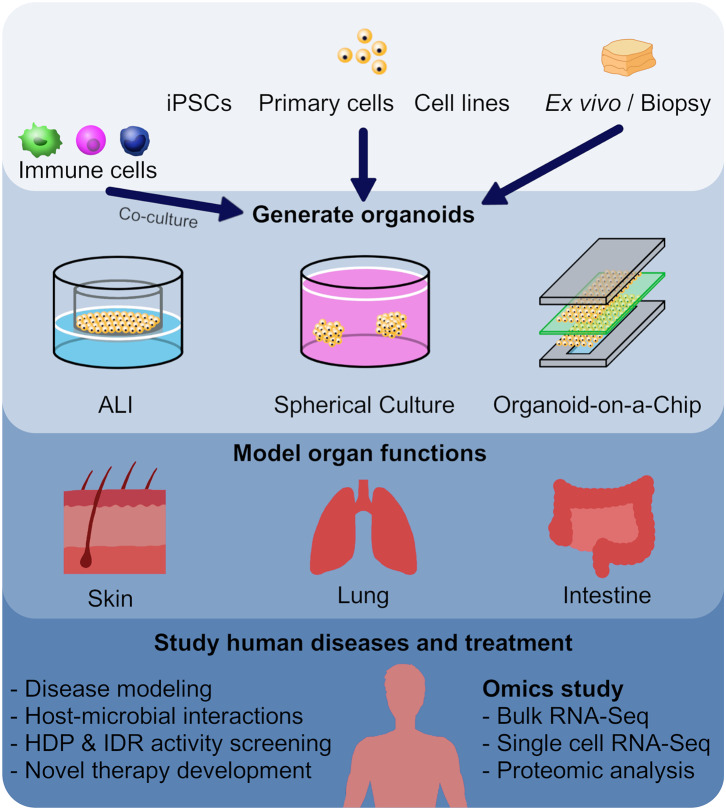
Utilizing organoid models as a screening method in the development of new host defense peptides. Human or animal induced pluripotent stem cells (iPSCs), embryonic stem cells, neonatal tissue stem cells, or *ex vivo* adult progenitors can all serve as starting materials to generate various organoids. In this review, we focused on skin, lung and intestinal organoids, which recapitulate the architecture, functions and multi-cellular components present in the tissue of origin. In general, there are three main forms of organoids: air-liquid interface (ALI) constructs, spheroids, and organ-on-a-chip models. These different forms of organoids, together with *omics* characterization, have provided mechanistic insights to diseases and host-microbial interactions, and provide novel tools for HDP and IDR screening.

Recent research has demonstrated that host defense peptides (HDPs) and its analogs innate defense regulator (IDR) peptides and peptidomimetics have strong potential to be developed into alternative/adjunctive therapies to fight against AMR organisms. They also address an urgent need for new ways of treating inflammation that underlies the pathology of almost every human disease. However, one of the biggest hurdles is the validity and translatability of current *in vitro* and animal model systems used for drug screening. In this review, we examined the use of human organoid systems focusing on skin, lung, and intestinal organoids for disease modeling and drug screening. Together with *omics* analyses, we will discuss the prospect of using organoid systems to aid in clinical translation of HDP research (Figure 1).

## Host Defense Peptide, Innate Defense Regulator, And Peptidomimetics As Alternative Therapies

HDPs, also known as antimicrobial peptides (AMPs), are naturally occurring cationic amphipathic polypeptides found ubiquitously in most species of life and play essential roles in providing protection against pathogens and modulating immunity (Hancock and Lehrer, 1998). To date, there are >3,000 HDPs described from the six kingdoms (animals, fungi, plants, and protists, with related molecules in bacteria and archaea): http://aps.unmc.edu/AP/main.php (Wang et al., 2016). These peptides tend to be relatively short (composed of ~12–50 amino acids), amphipathic, and have a net positive charge of +2 to +9 at physiological pH (Hancock and Sahl, 2006; Choi and Mookherjee, 2012). HDPs are an important component of the host immune system, participating in both innate and adaptive immunity (Hancock et al., 2016). They possess multifaceted biological functions in modulating host immune responses, including mediating immune cell recruitment and functions in part by regulating the production of cytokines and chemokines, suppression of inflammatory responses, enhancement of angiogenesis, and wound healing, etc. (Hancock et al., 2016). These host responses contribute to the resolution of infection and inflammation, which suggests that related synthetic IDR peptides may be excellent therapeutic candidates to treat infection and inflammatory diseases.

HDPs have broad-spectrum direct antimicrobial activities against Gram-positive and Gram-negative bacteria, viruses, fungi, and parasites (Ganz, 2003; Powers and Hancock, 2003; Straus and Hancock, 2006; De Zoysa et al., 2015). Several modes of actions had been proposed to explain antimicrobial effects of HDPs. Some of these mechanisms are directly targeting microorganisms to cause bactericidal effects, such as mediating damages to microbial cell membrane, inducing microbial DNA/RNA damages, and interacting with fungal mitochondria to cause cell lysis. While other mechanisms, such as inhibiting the synthesis of macromolecules and inhibiting enzyme activities leading to inhibition of bacterial cell growth, or mediate immune modulations of the hosts, contribute to bacteriostatic effects (Moravej et al., 2018; Haney et al., 2019; Lei et al., 2019). Many anti-biofilm HDP derivatives can target conserved stringent stress response leading to the degradation of the stringent response secondary-messengers guanosine pentaphosphate or tetraphosphate, which results in biofilm eradication and reduction in bacterial abscess formation (de la Fuente-Nunez et al., 2014; Mansour et al., 2016). These peptides can also work synergistically with conventional antibiotics (Pletzer et al., 2018). To date there are no HDP that have navigated through the clinical trial process to approval status, although peptides are clearly appropriate as medicines (Seo et al., 2012; Sachdeva et al., 2016; Mishra et al., 2017). Clear examples exist of HDP-like compounds (modified cationic amphipathic peptides) including the naturally-produced bacterial products polymyxin B, polymyxin E/colistin, and gramicidin S, that are used as over the counter medicines in triple antibiotic ointments, and for treatment of serious Gram-negative infections, wound infections, and genital ulcers, respectively (Costa et al., 2019). There are several peptides related to HDP in advanced clinical trials and most have been limited to topical application (Hancock et al., 2016; Costa et al., 2019). The reason why no HDP peptides have been approved to date is likely complex but may reflect issues such as selection of clinical targets, toxicity, low peptide stability, low bioavailability, and relatively high production costs (Seo et al., 2012; Mansour et al., 2014; Mishra et al., 2017). Some of these drawbacks have been addressed by creating synthetic analogs known as innate defense regulator (IDR) peptides and peptidomimetics using natural HDPs as templates. These synthetic analogs mimic the modes of action of the natural HDPs, but can be designed with enhanced stability, lower host toxicity, and lower cost of production (Hancock et al., 2016). Furthermore, advances in delivery systems and formulations, such as liposomes, polymeric nanoparticles, nanogels, cream, ointment, and wafers, can enhance the stability and bioavailability of HDPs (Piotrowska et al., 2017). To overcome peptide stability and high production costs, innovative chemistry and biotechnology allows the scale-up production of HDP and its analogs with lower synthetic costs (Bommarius et al., 2010; Ishida et al., 2016). For example, Vogel et al. successfully used calmodulin as a fusion partner to enhance the production of several HDP (e.g., melittin, fowlicidin-1, tritrpticin, indolicidin, lactoferrampin B, and human β-defensin 3) in *Escherichia coli* (Ishida et al., 2016).

With increasing interest in developing HDPs as alternative antimicrobial agents against AMR infections, there are researches addressing the potential for development of resistance to the antibacterial action of host defense peptides (Nizet, 2006; Cole and Nizet, 2016; Phoenix et al., 2016; Bechinger and Gorr, 2017). These have tended to show that for most peptides, resistance is very slow (over few 100 generations) to develop as compared to most conventional antibiotics (as little as one generation) (Cole and Nizet, 2016; Phoenix et al., 2016). Another consideration is that the direct killing effects of HDPs are drastically reduced in the presence of physiological concentrations of divalent cations and polyanions like the glycosaminoglycan heparin (Mansour et al., 2014). However, the ability to modulate host immunity to fight against infection without directly targeting the microbe gives HDPs an advantage in that this action occurs readily under physiological (tissue culture) conditions, and since it involves activation of host mechanisms does not induce bacterial resistance (Hancock and Sahl, 2006). This was exemplified by a recent study which showed that developing resistance to HDP in *Staphylococcus aureus*, evolved *in vitro* in the presence of HDPs, did not provide survival advantages to the bacteria in a host environment (El Shazely et al., 2019). In addition, there is increasing evidence that HDPs and their synthetic analogs are synergistic with conventional antibiotics, indicating that these peptides can also be use as adjunctive therapies (Rudilla et al., 2016; Wu et al., 2017). These unique characteristics have further shown that HDPs and their synthetic analogs are excellent candidates to be developed as alternative therapies against antibiotic resistant bacteria.

## Limitations of Using *in vitro* Immortalized Cell Lines And Primary Cells Models in Drug Discovery

Currently, one of the biggest hurdles in drug development is the translation of research breakthroughs from basic science to clinical trials. In general, antimicrobial drug development often begins by identifying and screening possible drug candidates using efficacy assays with diverse bacteria (including in the context of host cells in the case of intracellular pathogens), toxicity assays with specific cells or cell lines *in vitro*, followed by efficacy and toxicity tests in *in vivo* animal models, before proceeding to clinical trials. For anti-inflammatory agents, initial screens of both efficacy and toxicity would involve specific cell lines and/or primary cells. Each of the above steps are essential, but also include certain limitations, the most important of which are *in vitro* assays with isolated cells do not capture the complexity of the infectious/inflammatory milieu in the body, while animal models often are incomplete mimics of human diseases. Here, we briefly discuss some of the limitations of using immortalized cell lines and primary cells for drug screening.

Utilizing immortalized cell lines to study complex biological systems have been invaluable to expand our understanding of disease biology. The advantages of cell lines lie in their consistency, reproducibility, and unlimited supply (Kaur and Dufour, 2012). However, many disadvantages also plague cell lines. The process of immortalization often freezes the cell in an immature state, such that they have to be further activated, often in a specific and/or non-physiological manner. For example, human monocytic cell lines like THP-1, UA37, and HL-60 require activation with phorbol 12-myristate-13-acetate and/or 1 α, 25-dihydroxyvitamin D3 for macrophage maturation (Daigneault et al., 2010). In the case of studying the facultative intracellular pathogen *Salmonella enteric* sv*. typhimurium*, THP-1 differentiation was largely dependent on phorbol 12-myristate-13-acetate dosage and time administered to mirror the infection rates found in human monocyte derived macrophages (Starr et al., 2018).

The major disadvantage is that immortalized cells require transformation to enable them to continuously replicate, and thus their underlying biology is by definition different from the cells from which they are derived. For example, deviations in transcriptomic and proteomic expression are observed when comparing established hepatocyte cell lines to its related primary cell (Olsavsky et al., 2007; Pan et al., 2009). In fact, important features can be lost resulting in divergent cell lines. It is also well-established that genetic differences persist between immortalized cell lines and their equivalent primary tissues which hints at a lack of environmental complexity. Other confounding features of cell lines include genetic drift over the course of serial passages, the potential for contamination by other cell types and/or microorganisms and the misidentification of cell lines (Drexler and Uphoff, 2002; Capes-Davis et al., 2010). Mycoplasma contamination in particular has incidence rates reported to be as high as 35% for immortal cell lines (Drexler and Uphoff, 2002). Awareness of these differences is thus important when establishing host influences in drug screening, and in monitoring host-pathogen interactions that can affect, hinder or alter the potential for clinical applications.

To overcome these obstacles, primary cells can be used, as many of the morphological and functional features of host tissues are conserved. Although the process of isolating and differentiating of primary cells such as monocyte-derived macrophages (MDM) are well-established, there are some limitations in using primary cells. Compared to immortalized cell lines, obtaining pure populations of primary cells on a regular basis from different donors (or even repeatedly obtaining cells from the same donor) is relatively-speaking more difficult, especially when low abundance cells or cells from patients with rare diseases are required. In the case of tissue macrophages, circulating tumor cells, and vascular epithelial cells, appreciable yields are difficult to obtain (Gudjonsson et al., 2002). Other primary cells, such as neutrophils, have relatively short half-lives that limit their use in many assays (Hirz and Dumontet, 2016). Meanwhile, high biological variability amongst donors, low self-renewal ability, and the relative difficulty of genetic manipulation also pose obstacles and can limit some experimental outcomes.

## Limitations of Using *in vivo* Animal Models for Drug Discovery

Animal models are an invaluable part of drug development and have helped in the advance of innumerable discoveries in human biology and diseases (Brom, 2002; Festing, 2004). Animal models provide a more complex system that allows us to address a variety of scientific questions, from exploration of physiological functions and systemic interactions between organs and the immune system, to development and assessment of toxicity and efficacy of vaccines or therapies (Barre-Sinoussi and Montagutelli, 2015). Despite their immense impact, the use of animal models also has certain limitations, and results obtained from animal research are not always confirmed in human studies.

In particular, there are genetic and physiological variations between species and within the same species. For example, laboratory mice and rats, two of the most common animals used in the scientific world, are often inbred and have highly homogeneous genetic backgrounds within each strain (Mouse Genome Sequencing et al., 2002; Coors et al., 2010; Smith et al., 2014). The inherent differences between each strain contribute to their relative susceptibility or resistance to both induced and spontaneously occurring diseases. For example, depending on the genetic background of the mice, some strains are completely resistant to Ebola virus, while others are highly susceptible to the infection (Rasmussen et al., 2014). Another example showed that certain strains of mice with host resistance factor SLC11A1 (NRAMP1) are more resistance to infections by intracellular pathogens, such as *Mycobacterium avium* subsp*. Avium*, and *Salmonella* (Cunrath and Bumann, 2019). Secondly, impacts of biological pathways in animals and humans can be different (Muzio et al., 2000; Heinz et al., 2003). For example, mice deficient in galactose-1-phosphate uridyltransferase did not show any adverse effects or mimic human clinical symptoms when placed on a high galactose diet or after injections of galactose, even though these mice mimic galactosemia, a disorder that results from enzymatic deficiency of enzymes in galactose metabolism (Leslie et al., 1996; Elsea and Lucas, 2002). Ning and colleagues suggested that the lack of toxicity in galactose-1-phosphate uridyltransferase deficient mice was due to differences in galactose transport across the blood brain barrier between humans and mice (Ning et al., 2001). Similarly, the immune systems of mice and men vary substantially (Zschaler et al., 2014). For example, the critical chemokine that attracts neutrophils in man, IL-8, does not exist in mouse. Thirdly, the toxicity or the lack of certain molecules or chemicals in animals is not always reflected in humans. Analyses done by Baily and colleagues comparing drug responses between preclinical species (dog, rat, mouse, rabbit, and non-human primates) and man showed that the absence of toxicity in animal species was not predictive of an absence of adverse drug reactions in humans (Bailey et al., 2014, 2015; Bailey and Balls, 2019). For instance, vancomycin, often used as a last resort antibiotic against multi-drug-resistant Gram-positive bacteria, can cause hypersensitivity reactions such as the Red Man Syndrome and anaphylaxis, but these are only observed in man (Sivagnanam and Deleu, 2003). In addition, some toxicity and adverse effects only appear after long-term usage. For example, Vioxx (rofecoxib), a non-steroidal anti-inflammatory drug approved in 1999 by the US Food and Drug Administration for the prevention of recurrent colorectal polyps in colorectal adenomas patients, was found to induce an increased risk of cardiovascular morbidity and mortality in patients who took the drug for more than 18 months (Juni et al., 2004). Withdrawal of the drug was announced by Merck in September 2004, after an estimated 88,000 people suffered heart attacks, and 38,000 died as the result of taking Vioxx (Juni et al., 2004).

Another major consideration is in the accuracy of animal models in predicting efficacy. Animal models can provide immense amounts of information, that is presumed to be more indicative than *in vitro* assays of efficacy in man, but there is no single animal model that perfectly mimics a given human disease (Hainsworth et al., 2017). For example, animal models of infection are often short term, with animals dying within 24 h from the major AMR pathogens, while human infections usually occur over much longer periods of time. Similarly, it is difficult to precisely replicate polygenic genetic diseases such as asthma, inflammatory bowel disease, etc., and long-term environmental influences in human disease are almost impossible to replicate. In addition, overall animal models are very costly, time-consuming, and labor intensive, while posing ethical concerns (Rollin, 2003; Ideland, 2009; Coors et al., 2010). The most appropriate and informative animal models must be carefully selected considering the principles of the three R's (Reduction, Replacement, and Refinement) whenever possible (Hubrecht and Carter, 2019).

In recent years, there has been an increased push for the development of alternative strategies to reduce or replace animal experiments (Adler et al., 2011; Hay et al., 2014; Swaminathan et al., 2019). To emphasize the need for alternative models to be developed, a directive was signed in the USA in 2019 to prioritize efforts to reduce animal studies by 30% by 2025, and eliminate all mammal studies by 2035 (Agency, 2019). The recent surge in the development of human pluripotent stem cell and organoid systems can address some of the limitations mentioned above and bring us a step closer to the development of more translatable models for drug discovery, transplantation, and personalized medicine.

## Immune Cells Derived From Induced Pluripotent Stem Cells

In 2006, Takahashi and Yamanaka used a set of four transcription factors, Oct3/4, Sox2, c-Myc, and Klf4 to revert murine fibroblasts to a stem cell-like state, termed induced pluripotent stem cells (iPSC) (Takahashi and Yamanaka, 2006). With the same four transcription factors, this methodology was successfully applied to human dermal fibroblasts resulting in human iPSC (Takahashi et al., 2007). This discovery has spurred many groups to further refine the transcription factors needed to promote pluripotency in humans and murine somatic cells (Kim et al., 2008; Zhu et al., 2010; Wang L. et al., 2017). Chemical alternatives are also being interrogated to replace or enhance the stem cell-like qualities of adult mammalian somatic cells (Feng et al., 2009).

Macrophages, one of the main effectors of the immune system, have the ability to adopt different functional states depending on stimulus exposure. Originally described by Nobel Prize winner Elie Metchnikoff in 1908, macrophages were reported as phagocytic cells that engulf foreign invaders, leading him to name the process as phagocytosis (Kaufmann, 2008). Phagocytosis is a principal effector mechanism of innate immunity and is highly involved in the host's protective mechanisms against harmful pathogens. Metchnikoff's initial findings on the host defense and effector functions of macrophages have provided focus for much of the subsequent research on these cells. Monocyte functionality is diverse and encompasses features such as homeostasis, immune defense, and tissue repair (Ziegler-Heitbrock et al., 2010). The classification of macrophages is parallel to T-helper lymphocyte nomenclature with Th1 and Th2 lymphocytes, corresponding to classically activated (M1) and alternatively activated (M2) macrophages, although most authors consider that there is a broad spectrum of macrophage phenotypes spanning these two categories. The nomenclature is based on environmental stimuli, cellular phenotype, and the resulting physiology that separates macrophages into these broad categories. M1 are predominantly microbicidal macrophages with increased pro-inflammatory mediator levels, while M2 are macrophages with stunted inflammatory capacity and increased phagocytic activity and wound repair capabilities (Mosser and Edwards, 2008). Additional research has since further divided the M2 phenotype into three subsets: M2a, M2b, M2c (Tarique et al., 2015). Research from our lab has suggested that endotoxin tolerant macrophages (treated twice with endotoxin) are reprogrammed into an amnesic state that fails to recognize and respond to bacterial signatures, has characteristics similar to M2 macrophages and is predictive of the development of severe sepsis (Pena et al., 2011, 2014).

With the production of stem cell-like characteristics from adult somatic cells, many protocols have been established to differentiate these cells to specific cells of interest. Pluripotent iPSCs were generated with varying degrees of success depending on starting material and their associated conversion rates. In the case of human induced pluripotent stem cell derived macrophages (iPSDM), they have a strong genetic, functional, and transcriptomic similarity to monocyte derived macrophages (Hale et al., 2015). Furthermore, the generation of iPSDMs is scalable and therefore provides a source of unlimited genotype-specific cells that can potentially reflect the disease state from which the iPSCs are derived.

The continued characterization of myeloid development reports genetic distinctions between two cellular lineages. Each lineage originates from either the Myb-independent yolk-sac (YS) or Myb-dependent hematopoietic stem cells. The transcription factor Myb is required for YS-derived tissue resident macrophages (Schulz et al., 2012). Additional findings have reported that tissue resident macrophages like microglia and Kupffer cells are capable of self-renewal with minimal replenishment from hematopoietic stem cells (Hashimoto et al., 2013). Though there are broad similarities between iPSDMs and monocyte-derived macrophages (MDMs), iPSDMs develop in a Myb-independent manner and therefore may be more closely related to tissue resident macrophages when compared to macrophages derived from hematopoietic stem cells (Buchrieser et al., 2017).

Macrophages exhibit the features of plasticity and are famously known to respond to polarization factors that cause them to differentiate and exhibit a wide range of immune responses. Though many methods have been developed to generate macrophages from human iPSCs, the majority utilize similar differentiation steps. Dome-like iPSCs are maintained on a monolayer of irradiated murine feeder cells. Human iPSCs can then be converted into a three-germ-layer embryoid body (EB). Differentiation of the blastocyst-like EB can then be induced, upon the removal of recombinant human FGF2 plus the supplementation with IL-3 and M-CSF, to shed myeloid precursor cells into the surrounding medium. Enhanced concentrations of M-CSF result in the terminal differentiation of iPSDMs. These macrophages, established from iPSCs express a variety of macrophage markers that include CD11b, CD14, EMR1, and CD68 without or with limited SSEA-4 and OCT3/4 markers associated with undifferentiated stem cells (Hale et al., 2015). It has been established that iPSDMs can be polarized and express myeloid cell surface markers and secrete similar proteins compared to MDMs (Cao et al., 2019). Not only do macrophages generated from iPSCs have a strong genetic and functional similarities to host macrophages to better interrogate diverse disease states and infections, but their scalability enhances their use as potential drug screening tools (Hale et al., 2015).

Macrophages derived from human iPSCs established by Hale et al. have been harnessed to generate both wild-type and mutant varieties (Hale et al., 2015; Yeung et al., 2017, 2019). The generated iPSDMs were compared to blood monocyte derived macrophages and found to comparably phagocytize *Chlamydia* and generate very similar gene expression responses (Hale et al., 2015). Interestingly, iPSDMs had increased rates of infection with the knockout of IRF5 and IL10RA (Yeung et al., 2017), indicating that stimulation of these factors might counteract infection. iPSDMs also can serve as a genetic and drug screening tool by using guide RNA (gRNA) library methods. Yeung et al. utilized a mutant library of Cas9-expressing THP-1 cells and screened these for their inability to take up *Salmonella*. She consequently identified 183 loci that are required for *Salmonella* invasion of macrophages, each of which represented a potential target for host-directed therapy (Yeung et al., 2019). Such a strategy is easily translatable to iPSDMs since iPSCs are very genetically malleable. Overall, iPSDMs have the ability to mimic many of the features of MDMs and also provide insights into the host mechanisms that drive and promote *C. trachomatis* infections.

## Organoid Models

It is obvious that one cannot ethically perform whole organism infection model studies in man. Thus, it is of interest to create appropriate surrogate models for screening. One important approach utilizes organoids, which are self-organized, three-dimensional (3D) multi-cellular structures that resemble a miniature form of an organ. An organoid can be derived from human or animal iPSCs, embryonic stem cells (ESCs), neonatal tissue stem cells, or *ex vivo* adult progenitors (Clevers, 2016). Organoids recapitulate the crucial aspects of the architecture, functions and multi-cellular components present in the tissue of origin. In addition, organoids can be expanded and maintained over a long period of time while retaining genomic stability (Clevers, 2016). In general, there are three main forms of organoids: air-liquid interface (ALI) constructs, spheroids, and organ-on-a-chip models (Figure 1). Each model has their advantages and disadvantages, which will be discussed further below (Table 1).

**Table 1:** Summary of the advantages and disadvantages of various drug screening models.

**Features**	**Animal**	***Ex vivo*/biopsy tissue**	**Submerged** ***in vitro*** **culture systems**		**Organoid systems**		
			**Immortalized cell lines**	**Primary cells**	**2D (ALI)**	**3D (spherical)**	**Organ-on-a-chip**
Cellular complexity	Complete immune system	Localized multi-cellular responses	Lack complex multicellular interactions	Lack complex multicellular interactions	Complex multicellular interaction	Complex multicellular interaction	Complex multicellular interaction
Consistency	Biological variability	Can only be used once	Do not precisely mimic primary cells; Passage can alter function^a^	Can only be used once	Inconsistent when primary cells are used	Inconsistent when primary cells are used	Hard to achieve consistency
Human specific	No	Yes, human tissues	Yes	Yes, human cells	Yes, human cells	Yes, human cells	Yes, human cells
Patient specific	No	Yes, patient specific	No	Yes	Can be using primary cells	Can be using primary cells	Can be using primary cells
Ethical approval	Required	Required with donor consent	Not needed	Required with donor consent	Only for primary cells	Only for primary cells	Only for primary cells
Ease of acquisition	Easy to obtain from vendors	Limited supply^b^	Easy to obtain; Unlimited supply	Limited supply^b^	Primary cells limited^c^	Primary cells limited^c^	Primary cells limited^c^
Variability	High	High	Low	High	Depends on cell origin	Depends on cell origin	Depends on cell origin
Technical issues	Very Labor intensive^d^	Labor intensive	Easy to use and maintain	Easy to use but variable over time	Labor intensive^e^	Labor intensive^e^	Labor intensive^e^
Long term mainten-ance	Yes	No	Yes	No	Yes	Yes	Yes
Cost	Expensive	Cost effective	Cost effective	Cost effective	Less expensive	Expensive	Expensive
Genetics?	Can introduce mutations	No	Yes, can mutate	Difficult	Yes	Yes	Yes

a *Genetic manipulation is required to obtain immortality of cell lines may cause altered phenotypes functions, and responses to stimuli. Passaging of cell lines can induce genotypic and/or phenotypic variations over time*.

b *Donors can vary over time which contributes to difficulties to obtain cells and experimental variability*.

c *These methods can use cell lines, iPSC and ESC cells which are relatively easily obtained. Primary cells have issues with availability and donor variation*.

d *Requires complex techniques and specialized facilities*.

e *Technically demanding to maintain and in the case of 3D organoids and organ on chip technically difficult to develop systems*.

Originally, organoids were used in basic research to understand fetal development, cell assembly, and neoplasia. Driven by rapid developments in stem cell research, the availability of human progenitor cells, and the desire to refine, reduce or replace the use of research animals, there is increasing interest in the field of biological and medical research to utilize organoids to study toxicology, oncology, microbiology, regenerative medicine, and drug development (Engelhart et al., 2005; Davies, 2018). To date, a wide variety of organoids have been developed to represent different organs of the body, including skin, brain, lung, heart, stomach, liver, and intestine. Organoids can also be used to model various diseases, including cancer and infectious disease (Clevers, 2016; Heo et al., 2018). In addition, the pathogenic processes of chronic diseases related to genetic defects, such as cystic fibrosis (CF), can be recapitulated using organoid models (McCauley et al., 2017). Here, we will focus on examining the use of human skin, lung, and intestinal epithelial organoids, and current knowledge regarding the use of *omics* analyses with organoids.

### Skin Organoids

Human skin, as the largest organ of the body, forms a protective barrier that provides a first line of immune defense. The intensive crosstalk among epithelial cells, stromal cells, immune cells, and the skin commensal bacteria are essential for maintaining immune hemostasis in the skin (Pasparakis et al., 2014; Kabashima et al., 2019). Although animal models are widely used to study human immunological responses and to test the activities of novel therapeutics, there is concern about whether animal models can truly reflect human immunology (Bracken, 2009). Human and mouse skin have differences in their structure and cellular components. For example, human skin has a thicker dermis and less hair follicle density compared to that of the mouse skin (Pasparakis et al., 2014). In addition, Langerhans cells and CD8^+^ T cells are the major immune cell types in the epidermis of human skin, as compared to dendritic epidermal T cells which are more predominant in mouse epidermis (Mestas and Hughes, 2004; Pasparakis et al., 2014). Therefore, there is a great need for human systems to study human diseases. Three-dimensional skin organoid models are emerging tools implemented in many aspects of skin research such as the study of barrier properties (El Ghalbzouri et al., 2008; Danso et al., 2015), wound healing (El Ghalbzouri et al., 2004; Egles et al., 2010; van Kilsdonk et al., 2013), bacterial infection, biofilm formation, and screening of antimicrobial peptides (Holland et al., 2009; Shepherd et al., 2009; de Breij et al., 2012, 2018; Boekema et al., 2013; Haisma et al., 2013, 2014, 2016; van Drongelen et al., 2014; den Reijer et al., 2016), and inflammatory cutaneous diseases (van den Bogaard et al., 2014; Honzke et al., 2016; Hubaux et al., 2018).

Three main types of skin organoid models recapitulate the characteristics of human skin with fully stratified layers and a competent skin barrier, namely, *in vitro* generated ALI skin models, the *ex vivo* skin model and “skin-on-chip” technology. In ALI models, cells are grown on a permeable filter. Once the cells are confluent, media is removed from the apical side of the filter, allowing the cells to become exposed to air and initiate differentiation. ALI models can be built from human primary cells isolated from skin explants (de Breij et al., 2012; den Reijer et al., 2016; Haisma et al., 2016), cell lines [e.g., N/TERT cells (van Drongelen et al., 2014; Reijnders et al., 2015; Smits et al., 2017) or HaCaT cells (Springer et al., 2003; Engelhart et al., 2005)], keratinocytes or fibroblasts differentiated from iPSCs or ESCs (Guenou et al., 2009; Itoh et al., 2013), or primary cells nucleofected with siRNA targeting a gene of interest (Mildner et al., 2010; Honzke et al., 2016; Wallmeyer et al., 2017). N/TERT cells, for example, are immortalized by ectopic expression of the human telomerase reverse transcriptase gene while also losing the pRB/p16^INK4a^ cell cycle control mechanism (Dickson et al., 2000). The epidermal skin model contains about 8–10 epidermal cell layers with fully stratified layers (e.g., stratum corneum, stratum granulosum, stratum spinosum, stratum basale) expressing structural proteins (e.g., Keratin 10 and Keratin 16), tight junction proteins (e.g., claudin-1, claudin-4 and occludin), antimicrobial peptides (e.g., hBD-2, hBD-3, and RNAse7), and cytokines and chemokines (e.g., IL-8, IL-1α, IL-1β) (de Breij et al., 2012; Basler et al., 2017). In contrast, the full-thickness human skin equivalent has, in addition of the epidermis, a continuous basement membrane and fibroblast-seeded collagen or fibrin matrix (Holland et al., 2008; Haisma et al., 2013, 2014). Alternatively, epidermis can be established on top of a human decellularized dermal scaffold referred to as a de-epidermized dermis (Tjabringa et al., 2008; Shepherd et al., 2009; van Kilsdonk et al., 2013). The *in vitro* models mimic the origin and the genetic make-up of the epidermis and dermis in human skin. Immune cells such as Langerhans cells (Regnier et al., 1997; Facy et al., 2004; Ouwehand et al., 2011), dermal-type macrophages (Bechetoille et al., 2011), melanocytes (Gibbs et al., 2000), and CD4^+^ T cells (Engelhart et al., 2005; van den Bogaard et al., 2014; Wallmeyer et al., 2017), as well as endothelial cells (Ponec et al., 2004) and hair follicles (Hoeller et al., 2001) have been incorporated allowing the in-depth investigation of cellular responses.

The *ex vivo* human skin model is established by ALI culturing of human surplus skin samples (e.g., breast or abdominal skin) obtained from surgery with ethical approval and donors' consent (Ng et al., 2009; Danso et al., 2015; de Breij et al., 2018). *Ex vivo* skin can be trimmed into various thicknesses (typically 300 μm-3 mm) and maintained under laboratory conditions for about 2 weeks. This model provides normal skin structures and immune cells (e.g., CD14^+^ or CD1c^+^ monocytes/macrophages, CD11c^+^ DCs, CD56^+^CD3^−^ NK cells, CD4^+^ T cells, CD8^+^ T cells, and CD19^+^ B cells) (He et al., 2016), which reflect local immune responses and donor variability, but requires donor access and is difficult to genetically manipulate. Skin organoids can be thermally or physically wounded (Egles et al., 2010; Haisma et al., 2013, 2016) or injured by UV radiation (Bernerd and Asselineau, 2008) to create damaged skin models, which help the study of skin physiology and pathology with impaired skin barrier functions.

One major limitation of conventional ALI models is that these static culture systems make it difficult to study the dynamic processes such as nutrient exchange and immune cell migration. The microfluidic skin-on-chip technology helps to fill this gap since the skin is constructed in the presence of fluid flow under controlled microenvironments that closely mimics the mechanical force and biochemical gradients encountered by natural human skin (O'Neill et al., 2008; Wufuer et al., 2016; Lee et al., 2017). Monocytes (U937) have been co-cultured with HaCat keratinocyte-based epidermis in a bi-channel microfluidic setting for up to 17 days (Ramadan and Ting, 2016). Many skin-on-chip platforms contribute to drug development as biosensors can be implemented to provide non-invasive real-time tissue response readouts (e.g., membrane permeability and skin metabolism), which help in determining drug efficacy and toxicity (Wang Y. I. et al., 2017; Zhang et al., 2017; Alexander et al., 2018).

Skin models for cutaneous diseases such as atopic dermatitis and psoriasis have been employed to understand the initiation and progression of inflammatory skin diseases. Psoriasis and atopic dermatitis are prevalent chronic inflammatory skin diseases considered to be the paradigm of Th1/Th17 and Th2/Th22 types of disorders, respectively. One common approach for modeling disease symptoms is by stimulating skin equivalents with cytokines. For example, Tjabringa et al. discovered that skin equivalents stimulated with psoriasis-associated cytokines (IL-1α, TNF-α, and IL-6) displayed the molecular characteristics of psoriatic epidermis such as increased expression of skin-derived anti-leukoprotease (*SKALP*) and *hBD-2* (Tjabringa et al., 2008). Smits and colleagues established psoriasis and atopic dermatitis models using N/TERT epidermal skin by adding Th1/Th17 (TNF-α, IL-6, IL-1α, IL-17, and IL-22) cytokines and Th2 (IL-4 and IL-13) cytokines, respectively, during the final stage of the skin maturation (Smits et al., 2017). In the psoriasis model, Th1 and Th17 cytokines caused thickening of the stratum corneum, which was accompanied by decreases in the mRNA expression of epidermal differentiation proteins filaggrin (*FLG*) and loricrin (*LOR*), and upregulation in hBD-2 and SKALP protein expression (Smits et al., 2017). Supplementing skin equivalents with Th2 cytokines triggered signs of spongiosis in the superbasal layers of skin and apoptosis of basal cells, downregulated FLG, involucrin (IVL) and LOR, and upregulated the expression of the atopic dermatitis-related genes *CCL26* and *CA2* (Smits et al., 2017). Rouaud-Tinguely et al. demonstrated that supplementing epidermal skin equivalents with Poly I:C, TNF-α, IL-4, and IL-13 in the last 2 days of skin reconstruction, led to reproduction of the features of atopic dermatitis, including altered epidermal reconstruction and differentiation, enhanced thymic stromal lymphopoietin (TSLP) and IL-8 secretions (Rouaud-Tinguely et al., 2015). Transcriptomic analysis by microarray showed that comparing skin supplemented with the inflammatory cocktail to control skin, 3,816 genes were differentially expressed, which were enriched in 4 relevant pathways including cell adhesion, cell migration, inflammation, and epidermal cell differentiation (Rouaud-Tinguely et al., 2015).

An alternative approach for skin disorders modeling is by incorporation of immune cells. Engelhart et al. developed an eczematous dermatitis model by integrating activated CD45RO^+^ T cells into skin equivalents (Engelhart et al., 2005). This model reproduced several clinical hallmarks of eczematous dermatitis including T cell-induced keratinocyte apoptosis and impaired epidermal barriers, upregulation of intercellular adhesion molecule-1 and neurotrophin-4, and increases in the production of proinflammatory cytokines (IL-1α and IL-6) and chemokines (IL-8, IP-10, TARC, MCP-1, RANTES, and eotaxin) (Engelhart et al., 2005). In a study of atopic dermatitis pathophysiology, Wallmeyer et al. added activated human CD4^+^ T cells beneath the dermis of ALI skin culture. CD4^+^ T cells were able to exclusively migrate upwards in the skin equivalents generated from keratinocytes with *FLG* knockdown (*FLG*^−/−^*)*, and this process was directly stimulated by TSLP (Wallmeyer et al., 2017). Exposure of both *FLG*^+^ and *FLG*^−/−^skin equivalents to CD4^+^ T cells enhanced TSLP levels, which in turn shifted the polarization profile of activated CD4^+^ T cells from Th1/Th17 to Th2/Th22. This study highlights the critical role of TSLP in T cell migration as well as in the pathogenesis of atopic dermatitis (Wallmeyer et al., 2017). CD4^+^ T cell incorporation and migration in the skin model has also been used for studying psoriasis. Van den Bogaard et al. reported that skin equivalents populated with activated CD4^+^ T cells or *in vitro* polarized Th1 and Th17 cells induced the expression of psoriasis-associated marker genes *DEFB4, LCE3A, PI3*, and *KRT16* and elicited psoriasis-like epidermal inflammation (van den Bogaard et al., 2014). These cutaneous disease models provide excellent platforms for studying the immunomodulatory aspects of HDPs and to aid in their therapeutic development.

### Lung Organoids

Lung epithelium is the first point of contact with airborne pathogens, allergens, and pollutants. Airway lung epithelium is composed mainly of basal cells, ciliated cells, and secretory cells (club cells and goblet cells). Basal cells serve as progenitors for the ciliated and secretory cells (Shaykhiev, 2015). The ciliated cells contain beating cilia responsible for transporting foreign material trapped by the mucus out of the respiratory tract (Bustamante-Marin and Ostrowski, 2017). The secretory cells produce a thin layer of liquid containing mucin and glycoproteins, which entraps pathogens or allergens, and acts as lubricant to facilitate ciliary movement and moisten incoming air (Barkauskas et al., 2017; Bustamante-Marin and Ostrowski, 2017). The epithelium of the alveoli mainly consists of two cell types: alveolar type I cells (AEC1), which are responsible for gas exchange, and alveolar type II cells (AEC2) that secrete surfactants and proteins (Barkauskas et al., 2017). Under ideal conditions, where there are no airborne foreign materials, the turnover rate of lung epithelium is relatively low (Bowden, 1983; Kotton and Morrisey, 2014). However, in the presence of foreign materials or cellular injury, basal cells quickly proliferate and differentiate into one or more cell types to regenerate the epithelium and restore barrier function (Shaykhiev, 2015). Interestingly, when basal cells were selectively killed off via genetic modification, researchers have discovered that club cells were able to undergo reprogramming to become Krt5^+^Trp63^+^ basal cells and fill in the role (Tata et al., 2013; Pardo-Saganta et al., 2015). This robust flexibility and the ability to self-regenerate and repair the epithelium has sparked interest in the field of regenerative medicine and biological sciences, enabling a search for cell-based therapies or drug targets that can induce regeneration and repair damages induced by infection or chronic airway diseases. However, the relevance and validity of using traditional submerged *in vitro* cell lines (such as HBE16o^−^, Calu-3, A549) for toxicity and drug efficacy assessment is highly questionable (Frohlich and Salar-Behzadi, 2014; Clippinger et al., 2016). In addition, submerged *in vitro* models lack the ability to produce the physiological features of airway epithelium. Due to substantial differences in lung anatomy, physiology and molecular mechanisms between human and mice, results from *in vivo* animal model, especially toxicity studies, cannot be translated to man (Wright et al., 2008; Landsiedel et al., 2014). In addition, many animal lung disease models, such as CF, chronic obstructive pulmonary disease (COPD) and cancer, fail to sufficiently recapitulate the human symptoms of the diseases (Mortaz and Adcock, 2012; Kim et al., 2019). Furthermore, certain behaviors of laboratory animals, such as mice being obligate nose-breathers, can hinder the translatability of results to man (Benam et al., 2016). Thus, there is a need for an *in vitro* lung model that can recapitulate human lung physiology. The development of lung organoids has provided models that more closely recapitulate human lung physiology.

Lung organoids can be derived from both healthy and diseased primary lung cells (such as human bronchial epithelial cells, HBEC), ESCs or iPSCs (Barkauskas et al., 2017; Nikolic et al., 2017; Yamamoto et al., 2017; Miller et al., 2019). In order to form organoids, progenitors have to undergo stepwise directed differentiation, using culture medium with defined growth factor cocktails that activate and inhibit specific signaling pathways (Huang et al., 2015; Gkatzis et al., 2018). Briefly, human iPSCs are first differentiated into definitive endoderm by using high concentrations of activin A, followed induction of anterior foregut endoderm using Noggin to inhibit bone morphogenetic protein (BMP), as well as transforming growth factor-β (TGF-β) and activation of Wnt signaling (Huang et al., 2015; Mou et al., 2016). Subsequently, patterning into ventral anterior foregut fate by applying Wnt, BMP, fibroblast growth factor (FGF), retinoic acid and GSK3β antagonist CHIR9901, ultimately gives rise to lung and airway progenitors. Finally, these progenitor cells are subjected to maturation into airway and lung epithelial cells using Wnt, FGF, cAMP, and glucocorticoid agonism. The matured organoid consists of functional ciliated cells with beating cilia, basal cells (TRP63^+^KRT5^+^), and goblet cells (MUC5B^+^) (Huang et al., 2015; Hild and Jaffe, 2016). Using RNA-Seq, Dye and colleagues showed that the gene expression activity of the developed lung organoids resembles that of a developing human fetus. Chen and colleagues developed an improved technique to further induce the matured lung and airway progenitors into branching airway, which form alveolar-like structures in a matrigel 3D culture that expresses alveolar epithelial cell markers (Chen Y. W. et al., 2017). This model was further used to show that the organoids were able to recapitulate the *in vivo* characteristics of human lung development and enabled studies of respiratory syncytial virus infection and pulmonary fibrotic lung disease (Chen Y. W. et al., 2017). Recently, Sachs and colleagues demonstrated a method that allows long term expansion of pseudostratified airway organoids (Sachs et al., 2019). They used the model to assess a variety of pulmonary diseases: (1) organoids derived from CF patient progenitors cells were used to examine the function of cystic fibrosis transmembrane conductance regulator (CFTR); (2) Lung tumoroid were made to enable assessment of histopathology, gene mutation and drug screening; and (3) infected organoids were shown to recapitulate the central diseases features of respiratory syncytial virus infection, showing that neutrophil recruitment occurred during co-culture (Sachs et al., 2019). Yamamoto et al. performed long-term expansion of alveolar organoids containing alveolar epithelial type II (AEC2) derived from human iPSCs (Yamamoto et al., 2017). Using single-cell RNA-Seq, the authors showed that these alveolar stem cells have heterogeneous gene expression patterns that mimic lung cells from the late gestational phase rather than adult phase (Yamamoto et al., 2017). Furthermore, even though these alveolar organoids contained representative stem cell markers and mature lamellar bodies, they lacked MHC class II gene expression, indicating that they might require interaction with immune cells such as macrophages for further maturation (Yamamoto et al., 2017). However, transcriptomic analyses of these organoids treated with two drugs known to impact on AEC2 cells, namely amiodarone and GNE7915, provided putative mechanistic insights as to how these two drugs differentially induce toxicity (Yamamoto et al., 2017). In addition, several reports have shown that organoids derived from primary HBEC or patient specific iPSC maintain the patient's genetic and phenotypic signatures *in vitro* (Gras et al., 2012; Kim et al., 2019; Leibel et al., 2019). Thus overall, the spherical culture is a promising tool to study lung development and can be used to model a variety of chronic and infectious diseases. However, the interiorized lumen (facing inwards rather than outwards) makes stimulation and sample collection difficult. This limitation can be overcome with the use of an ALI model.

In ALI models, iPSC derived or primary lung epithelial cells are grown to confluence on a permeable filter (Upadhyay and Palmberg, 2018), and media then removed from the apical side of the filter. This exposes the cells to air and initiates differentiation into a matured, polarized pseudo-stratified epithelium, consisting of functional basal cells, ciliated cells, and secretory cells. The validity of the ALI lung model is supported by many studies that compare its structures, functions, and genetic profiles with nasal or bronchoscopically-obtained tracheal and bronchial brushings from human airways (Dvorak et al., 2011; Pezzulo et al., 2011; Fulcher and Randell, 2013). For example, using a combination of ALI model and mass spectrometry-based approaches, Kesimer et al. showed that human tracheobronchial epithelial cells grown in the ALI system shared 84/186 similar secretory host defense proteins with human tracheobronchial normal induced sputum (Kesimer et al., 2009). The ALI model has both apical (top) and basal (bottom) chambers, which allows for the convenient addition of any stimulus, as well as sample collection. The ALI model provides a convenient platform for determining epithelium integrity, by measuring the trans-epithelial electric resistance, mucociliary clearance, and cilia beat frequency (Gras et al., 2012; Gamez et al., 2015). To further assess the dynamic mechanical and biochemical microenvironment of the lung, the lung-on-a-chip model can be used. Lung-on-a-chip or airway-on-a-chip are microfluidic devices on microchips, consisting of a 3D cell culture system separated by a porous membrane, containing canals that allow continuous perfusion to mimic circulation in the body, and vacuum chambers to mimic breathing in human lungs (Konar et al., 2016). Various microsensors within the microchip enable the collection of real-time data, such as barrier function, surfactant production, protein production, fluid pressure, and cellular migration (Bhatia and Ingber, 2014). These organ-on-a-chip models are able to recapitulate *in vivo*-like environments and allow for the comparison of biological responses under normal and disease conditions (Benam et al., 2016). For example, Benam and colleagues used bronchiolar cells from normal or COPD patients to build airway-on-a-chip models to perform a matched study on electronic cigarette smoke-induced pathophysiology, such as oxidative stress and ciliary function (Benam et al., 2016). By combining *omics* analyses, they were able to identify 335 differentially expressed genes in normal smoking chips compared to non-smoking chips and 276 differentially expressed genes in COPD smoking chips compared to non-smoking chips, of which 147 were COPD specific and 129 were shared with smoke-exposed normal chips (Benam et al., 2016). This study showed the possibility of using a relatively “simple” organ-on-a-chip model to identify disease-specific responses and molecular signatures that might reveal novel potential therapeutic targets or diagnostic biomarkers.

### Intestinal Organoids

The intestinal epithelium is composed of a layer of epithelial cells that serve to facilitate digestion and absorption of nutrients, and is in constant contact with dietary antigens and diverse microorganisms (Turner, 2009). Human intestinal epithelium is comprised of multiple cell types, such as enterocytes, goblet cells, Paneth cells, and neuroendocrine cells. Enterocytes are columnar cells that are responsible for absorbing nutrients and secreting immunoglobulins (Kong et al., 2018). Neuroendocrine cells acts as chemo-receptors that detect the presence of microorganisms or dietary antigens, and initiate the appropriate responses by releasing hormones or peptides into the blood stream (Cox, 2016). Goblet cells are responsible for secreting mucins, which provide lubrication for food passage and create a barrier to prevent bacterial invasion (Hansson and Johansson, 2010). Paneth cells can sense the presence of bacteria through Toll-like receptor (TLR) activation, synthesize antimicrobial peptides and proteins, and provide signals to support intestinal stem cell self-renewal (Bevins and Salzman, 2011). Microarray and proteomic studies showed that goblet or Paneth lineage-specific intestinal organoids recapitulate the functions and phenotypes of their *in vivo* counterparts (Yin et al., 2014; Luu et al., 2018). Furthermore, by comparing label-free quantitative proteomic data from goblet or Paneth cell-enriched organoids with the Human Protein Atlas, novel markers for goblet and Paneth cells were identified (Luu et al., 2018). Transcriptomic profiling of intestinal epithelial cells and enteroids also provided insights into the spatial compartmentalization of TLRs and unique TLR-induced responses in the intestinal epithelium (Price et al., 2018). Finally, single-cell RNA-Seq of intestinal organoids has identified a rare population of hormone-producing enteroendocrine cells that express *Reg4* (Grun et al., 2015).

The intestinal epithelium possess a very high rate of self-renewal (every 4–5 days), where new intestinal epithelial cells arise in the crypt from intestinal stem cells (ISC) expressing leucine-rich-repeat-containing G-protein coupled receptor 5 (Lgr5), migrate up the crypt-villus axis, and differentiate into various mature epithelial cell subsets (Clevers, 2013). Due to the constant assaults from interacting with pathogens and foreign antigens, the maintenance of proper integrity, function, and immune responses of the intestinal epithelial is essential to prevent disease development. In order to understand the various intestinal diseases, host-microbe relationships, development and pathologies of the intestinal tract, researchers have utilized various *in vitro, ex vivo*, and *in vivo* models over the years.

Several intestinal submerged *in vitro* and *ex vivo* models have been developed using transformed carcinoma cell lines, and human fetal or germ-free rodent primary cells (George et al., 2019). However, the use of cell lines and primary cell models often encounters several limitations including: (1) lack of mature epithelial cell types and reduced genomic stability; (2) cell lines usually contain one type of cell that does not reflect the composition of intestinal epithelium, which is composed of multiple cell types; (3) cell lines do not produce mucin, which is an important host defense mechanism of the intestine; (4) cell lines tend to proliferate until sub-confluency, while *in vivo* intestinal epithelial cells proliferate to confluency essential for maintenance of epithelium integrity; (5) primary cells and *ex vivo* models are highly variable, expensive, and can only be maintain within a very short period of time; (6) species-specific issues of using non-human cell lines; and (7) cell line models lack the presence of microbiome. Studies showed that interaction between intestinal epithelium and commercial microflora in the gut is an essential aspect of health (Cornick et al., 2015; Jandhyala et al., 2015; Valdes et al., 2018; George et al., 2019). Alternatively, there are also many animal models that have been developed over the years to study intestine-related diseases, such as pathogen gastrointestinal infections and inflammatory bowel disease (IBD). Some examples of *in vivo* models include, but are not limited to ligated loops of animal intestines, chemically induced experimental intestinal injury and intestinal xenograft in immune-deficient mice. The *in vivo* models contain many of the essential cellular subsets, the microbiome and a more “complete” immune system, which allows the exploration of intestinal functions and diseases. Also, mouse and rat intestinal development is fairly similar to human intestine, while non-human primates have an even high degree of genetic (90%) and physiological similarity to human (Mouse Genome Sequencing et al., 2002; Coors et al., 2010; Smith et al., 2014). However, there are still many limitations in that: (1) non-human primates are expensive, invoke substantial ethical considerations, and have a potential hazard of contracting with zoonotic agents (Ideland, 2009; Coors et al., 2010); (2) harsh chemicals or genetic modification used in animal models, usually induce phenotypes similar, but not identical, to human intestinal diseases; and (3) differences in species-specific expression of Toll-like receptors affects the responses to various stimuli (Muzio et al., 2000; Heinz et al., 2003; Low et al., 2013). Due to these limitations, especially from a translational perspective, there is a need for human models that more closely reflect human intestinal diseases.

In this regard, intestinal organoids can provide a more representative culture system for the study of intestinal development, physiology, and diseases. Intestinal organoids, enteroids, or colonoids, can be formed from iPSCs or adult ISC (George et al., 2019). Enteroids contain various differentiated epithelial cell types that mimic small intestinal architecture, while colonoids consists of epithelial cell subsets similar to those found in large intestine or colon (Luu et al., 2018; Attili et al., 2019; In et al., 2019; Jones et al., 2019). The iPSC-derived organoids require meticulous maintenance, while adult ISC-derived organoids are established based on tissue of the intestinal crypts, obtained from surgery or endoscopic biopsies, but require relatively less maintenance (George et al., 2019). Briefly, the isolated Lgr5^+^ ISCs are embedded in Matrigel (a gelatinous protein mixture secreted by mouse sarcoma cells), with culture medium containing small molecules and growth factors (Wnt agonist R-spondin1, the bone morphogenetic protein (BMP) antagonist Noggin, and epidermal growth factor (EGF), Wnt3a, gastrin, progstaglandin E2, nicotinamide, the TGF-β receptor inhibitor A83-01 and p38 inhibitor SB202190) required for proliferation, differentiation and self-organize into a 3D organoid (Nanthakumar et al., 2000). Once formed, the structure of the organoid resembles normal intestinal epithelium, comprising of multiple crypt-like domains that contains villi, Paneth cells, goblet cells, and enterocytes (Sato et al., 2009; Yin et al., 2014). With proper maintenance, both types of intestinal organoids can retain the gene expression profile, function, and pathological characteristics of the normal intestine and have the ability to self-renewal, even after many passages (Sato et al., 2009; Middendorp et al., 2014; Suzuki et al., 2018). A 3D organoid can capture patient-specific *in vivo*-like complexity and architecture, making it an ideal model to study the mechanisms of tissue homeostasis and disease development for diseases such as ulcerative colitis or colorectal cancer (Otte et al., 2004). CRISPR/Cas9 technology can be used in conjunction with organoids to study many genetic gastroenterological disorders or to screen anticancer candidates in different stages of tumorigenesis (Cario and Podolsky, 2000; Otte et al., 2004). Pathogens, such as *Vibrio cholera*, enteropathogenic *Escherichia coli* and rotavirus, can be microinjected into the lumen of the organoid to study host-microbe interactions (Dotan et al., 2007; Cho, 2008; Spence et al., 2011; Dedhia et al., 2016). Furthermore, each organoid line can be expanded, frozen, and revived for multiple uses, which can potentially be use as biobanks (van de Wetering et al., 2015).

Although both the organoid and ALI have many advantages over conventional *in vitro* and *in vivo* models, both still lack other supporting cells, immune cells and physiological luminal and blood flow. In addition, spherical organoids have an enclosed lumen, which makes the process of stimulation and sample collection difficult (Bhatia and Ingber, 2014). To overcome these hurdles, researchers have developed a microfluidic Organ Chip model for the human intestine, also known as gut-on-a-chip, where microchannels are lined with human microvascular endothelium, immune cells or bacteria. In addition, culture medium or stimuli can be perfused through each microchannel with a syringe or peristaltic pump (Vickerman et al., 2008; Bhatia and Ingber, 2014).

Given that a number of severe human gut diseases, such as Crohn's disease or salmonellosis are associated with Paneth cell dysfunction, Jones et al. used the integration of various *omics* data from Paneth cell-enriched enteroids to investigate the role of autophagy in these pathologies (Jones et al., 2019). Specifically, previous genome-wide association studies identified a mutation in the key autophagy gene, *ATG16L1*, that prevent autophagy-mediated defense against bacterial pathogens. By comparing the quantitative proteome of Paneth cell-enriched enteroids from mutant *ATG16L1* with that of wild-type cells, the authors showed an increase in protein abundance in *Atg16l1-*deficient cells due to autophagy impairment (Jones et al., 2019), as confirmed by protein-protein interaction network analysis. Furthermore, integration of transcriptomic and proteomic data, together with functional enrichment and network analysis showed that these autophagy impaired *Atg16l1-*deficient organoids had reduced exocytosis-mediated secretion of antimicrobial peptides (Jones et al., 2019).

Intestinal organoids also serve as an important model for characterizing host-microbe-interactions. For example, characterization of *Salmonella enteric* sv*. typhimurium* interaction with the intestinal epithelium has been challenging using whole mouse infection models due to rare epithelial interactions in the unligated intestine. Similarly using traditional immortalized intestinal cell lines is difficult as they lack the different cell types and architecture of a typical intestinal epithelium. However, microinjection of *S. typhimurium* into intestinal organoids generated from human iPSCs provided an opportunity to understand how *Salmonella* interactions with different intestinal cell types impact on its ability to spread. Transcriptomic analyses by RNA-Seq of intestinal organoids upon *S. typhimurium* infection identified the upregulation of genes, including those encoding interleukins and proinflammatory cytokines, in response to *Salmonella* infection (Forbester et al., 2015). Furthermore, goblet cell-associated genes such as GCNT3 and MUC2 were also upregulated indicating that multiple cell types in the enteroids can respond to *Salmonella* infection (Forbester et al., 2015). The direct modeling of wild-type *S. typhimurium* and the intestinal cells of enteroids was further confirmed by microscopy and required *S. typhimurium* invasion protein InvA (Forbester et al., 2015). These enteroids also were used to demonstrate the importance of alpha-defensins in restricting *S. enteric* sv. *typhimurium* (Wilson et al., 2015). Similarly, intestinal organoids can serve as a useful experimental system to characterize host transcriptional responses to microbiota-produced short-chain fatty acids (Lukovac et al., 2014).

## Discussion: Future Perspectives of Utilizing IPSC Derived Cells and Organoid Models in Screening for EffectivE HDPs

Currently, newly developed drug treatments for human diseases face limitations due to individual differences between patients, thus making it challenging to predict therapeutic outcomes and effectiveness. However, organoids based on a specific disease or a variety of individual scan potentially revolutionize precision therapy by improving disease modeling and drug screening to improve gene-drug associations (Bartfeld and Clevers, 2017; Takahashi, 2019). Below, we will discuss how various iPSC-derived immune cells and organoid models have been utilized in drug discovery and their potential use for screening HDPs.

### Induced Pluripotent Stem Cells-Derived Immune Cells

Infections from the obligate intracellular bacterium *C. trachomatis* is the leading cause of bacterial sexually transmitted diseases (STDs) and preventable blindness, and are a global health burden (Pinsent and Gambhir, 2017; Rowley et al., 2019). Chlamydial infections cause chronic disease and often are asymptomatic. If left untreated, STD infections can progress to the upper genital tract and result in pelvic inflammatory disease (PID) and infertility (Moodley et al., 2002). The complexity of chlamydial infections is in part, due the biphasic lifestyle of the infecting bacterium. Infectious, non-growing extracellular elementary bodies infect cells leading to differentiations into non-infectious, replicating intracellular reticulate bodies and are able to reside within infected macrophages (Gracey et al., 2013; Elwell et al., 2016; Yeung et al., 2017). Studying the host-pathogen interactions of M0, M1 or M2 polarized murine macrophage infected with *C. muridarum* demonstrated the susceptibility of M0 and M2 macrophages, and resistance of M1 macrophages, to infection (Gracey et al., 2013), as also shown for *C. trachomatis* (Yeung et al., 2017). While internalized, *Chlamydia* spp. survival and proliferation is favored in M2 polarized macrophages (Buchacher et al., 2015). Alternatively, classically activated M1 macrophages secrete high levels of proinflammatory cytokines and exhibit antimicrobial effects. Since *Chlamydia* only replicates in human cells, iPSDM (and possibly organoid models) would appear to be ideal for studying the mechanisms of infection and screening for anti-*Chlamydia* therapeutics, including host directed therapies (Yeung et al., 2017).

Unlike many pathogenic bacteria, antibiotic resistance is a rare occurrence with *Chlamydia* spp. Like antibiotics, some HDPs can have robust antimicrobial effects. The 21-residue synthetic peptide, Pep1 demonstrated direct antimicrobial activity against intracellular *Chlamydia*. Application of Pep-1 to infected macrophages resulted in complete eradication of chlamydial inclusions while also blocking growth of infectious progeny (Park et al., 2009). The only human cathelicidin, a C-terminal 37 amino acid LL-37 also demonstrated both antimicrobial activity and immune modulation. *C. trachomatis* has a highly conserved cryptic plasmid which encodes eight open reading frames. Of the eight, virulence factor Pgp3 (pORF5) is the only protein secreted into chlamydial inclusions and the cell cytosol and then binds to Li et al. (2008) and inactivates (Hou et al., 2015) cathelicidin human HDP LL-37.

**Figure 2:**
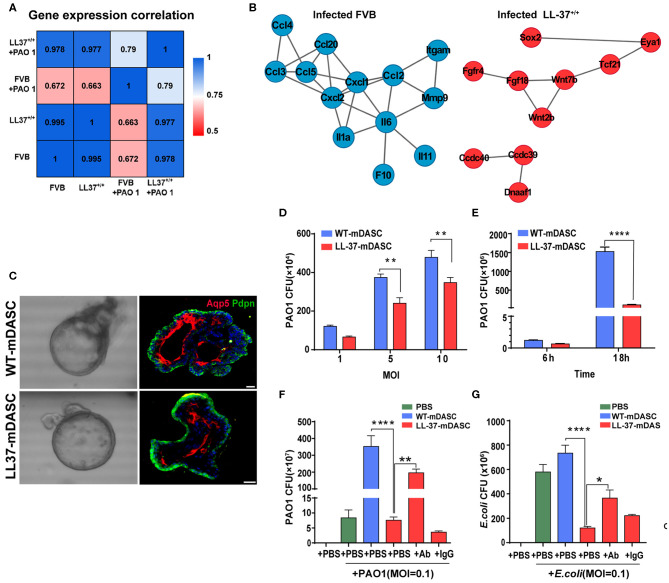
Genetically engineered distal airway stem cell transplantation protects mice from pulmonary infection. **(A)** Heatmap showing transcriptome profile correlation values of indicated lung tissue samples before and after PAO1 infection. **(B)** Protein-protein interaction network of selected genes with high expression level in PAO1-infected wild-type lung (blue) and PAO1-infected LL-37^+/+^ lung (red), respectively. **(C)** Representative 3D organoid culture of mDASCs with expression of type I alveolar cell markers (Aqp5 and Pdpn). Left panels, bright-field imaging of 3D organoids. Right panels, immunofluorescence of organoid sections. Scale bar, 20 μm. **(D)** Co-culture of bacteria with DASCs shows antimicrobial effects in dose-dependent manner. Initial additions of PAO1 were 0.1 ×, 0.5 × and 1 × 10^4^ CFU, respectively. Co-culture duration, 6 h. *n* = 4. Error bars, SEM. MOI, multiplicity of infection. **(E)** Co-culture of bacteria with DASCs shows antimicrobial effects in time-dependent manner. Initial concentration of PAO1 was 1 × 10^4^ CFU. MOI = 1. *n* = 3. Error bars, SEM. **(F,G)** Preincubation of cells with anti-LL-37 antibody, but not IgG control, significantly reduced anti-PAO1 **(F)** and anti-Escherichia coli **(G)** effects of LL-37-mDASCs. Initial dose of bacteria was 10^3^ CFU. Co-culture duration, 18 h. *n* = 4 in **(F)** and *n* = 3 in **(G)**. Error bars, SEM. Statistics for graphs: two-way ANOVA followed by Sidak's test **(D,E)** and one-way ANOVA followed by Tukey's test **(F,G)**. *P < 0.05; **P < 0.01; ****P < 0.0001. These results were from Zhou et al. (2019), published under the terms of the CC by 4.0 license. Full terms at https://creativecommons.org/licenses/by/4.0/. This figure combined Figures 2, 3 from the original article with permission from the corresponding author.

Two of the potential limitations in the use of HDPs as anti-infection or anti-inflammatory therapy are peptide instability and off-target toxicity (Johansson et al., 1998; Sieprawska-Lupa et al., 2004; Heilborn et al., 2005; Vandamme et al., 2012; Mishra et al., 2017; Zhou et al., 2019). To address these concerns, Zhou and colleagues proposed to use stem cell-based gene delivery therapy with the human HDP LL-37 to provide local protection against pulmonary infections (Zhou et al., 2019). The HDP peptide LL-37 is known to have a broad range of antimicrobial and immunomodulatory activities that provides protection against infections (Vandamme et al., 2012; van der Does et al., 2012). A transgenic mouse strain was constructed that constitutively expressed LL-37 (LL-37^+/+^) to confirm the *in vivo* protective effects of the peptide against *P. aeruginosa* PA01 infection (Zhou et al., 2019). Using RNA-Seq, they compared the transcriptomic profiles of wild-type and LL-37^+/+^ mice before and after PA01 infection (Figure 2). In contract to the wild-type mice, the transcriptomic profiles of LL-37^+/+^ mice before and after PA01 infection were very similar, indicating that LL-37^+/+^ mice were protected from PA01 challenge-induced alterations. In response to PA01 infection, WT mice revealed an inflammation driven protein-protein interaction network whereas a lung development-related molecular network was found in LL-37^+/+^ mice, indicating a protective role of LL-37 expression in mouse lung (Figure 2). Secondly, Zhou et al. introduced the LL-37 gene into mouse P63^+^/Krt5^+^ distal airway stem cells (DASC) by lentiviral transduction to create mDASC that constitutively expresses LL-37 (LL-37-mDASC). Using a 3D organoid system, they showed that the genetically engineered LL-37-mDASC were able to form alveolar-like sphere structures, consisting of differentiated cells expressing type I alveolar cell markers, and were able to secrete functional LL-37 that inhibited growth of both PA01 and *Escherichia coli* (Figure 2). In a lung injury mouse model, transplanted LL-37-mDASCs incorporated only into the injured lung tissues, but not healthy lung tissues, which suggested that LL-37-mDASC specifically targeted injured lung tissues. The regenerated LL-37 lung, containing LL-37-mDASC, enhanced bacterial clearance after intratracheal challenge with either PA01 or *E. coli*. By using a combination of genetically engineered stem cells, animal and organoid models and RNA-Seq analyses, Zhou and colleagues showed that genetically engineered DASCs, which constitutively express the human HDP LL-37, can provide protection against pulmonary infections and enhance the repair of lung injury (Zhou et al., 2019).

### Skin Models

**Figure 3:**
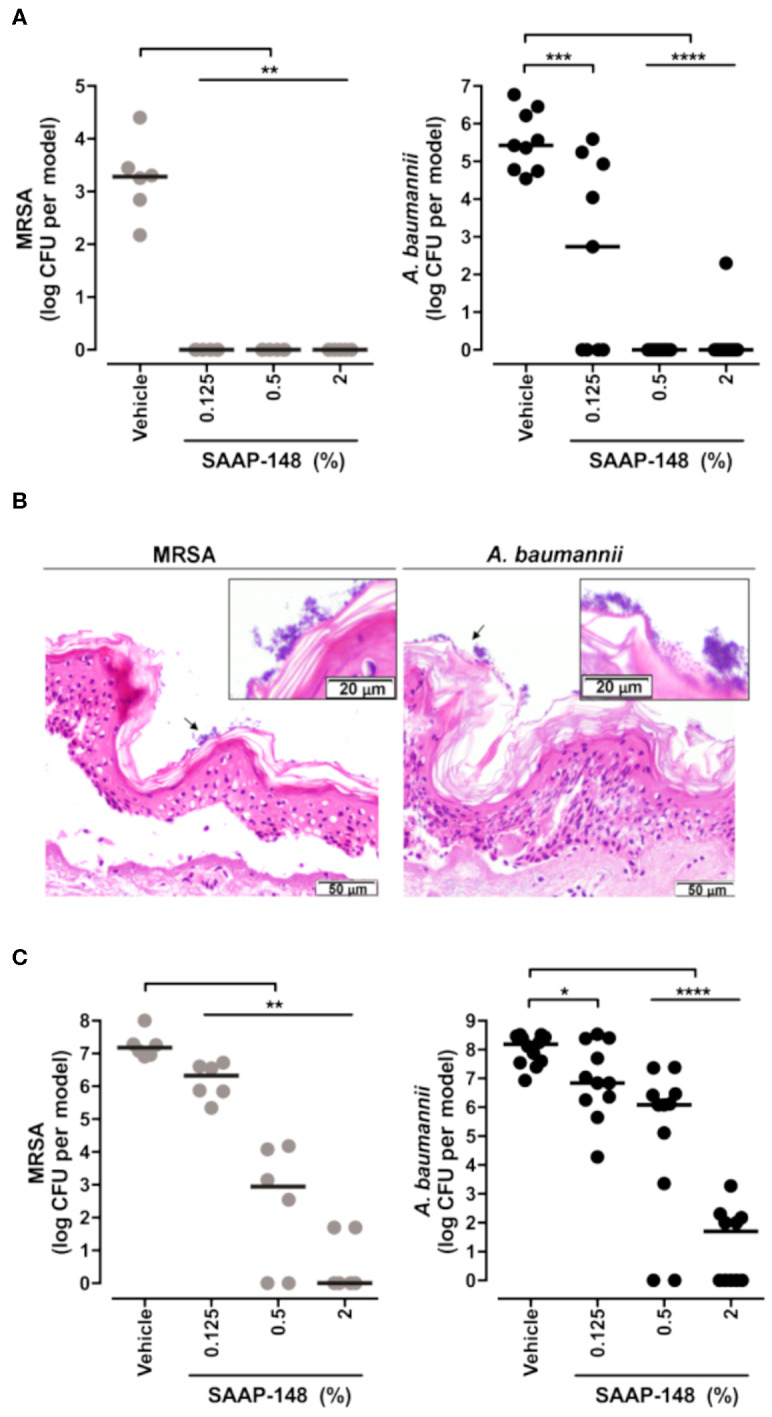
Topical application of SAAP-148 ointment eradicates acute and established infections of MRSA and *A. baumannii* from the skin. Use of an *ex vivo* human skin ALI model to demonstrate that topical application of peptide SAAP-148 ointment eradicates acute **(A)** and established **(C)** infections of methicillin resistant *Staphylococcus aureus* (MRSA) and *Acinetobacter baumannii* infections from the skin within 4 h of treatment. Results are expressed as the numbers of viable bacteria (in log_10_ CFU) per skin model of three to six donors. Each circle represents one skin sample, and bars indicate medians. *Significantly different (*P < 0.05, **P < 0.01, ***P < 0.001, and ****P < 0.0001) as compared to the vehicle, as calculated using the Mann-Whitney rank sum test. **(B)** shows that these organisms form biofilms on the skin surface 24 h after inoculation. These results were excerpted from de Breij et al. (2018), with permission from AAAS.

Researchers have employed skin models to investigate host-microbial interactions including skin commensal organisms such as *Staphylococcus epidermidis* and *Propionibacterium acnes* (Holland et al., 2008, 2009), and pathogens that are the leading cause of skin and soft tissue infections including *Staphylococcus aureus* (Charles et al., 2009; Shepherd et al., 2009; Haisma et al., 2013, 2014, 2016; van Drongelen et al., 2014; den Reijer et al., 2016; de Breij et al., 2018) and *P. aeruginosa* (Charles et al., 2009; Shepherd et al., 2009; Boekema et al., 2013). In the study of antimicrobial and immunomodulatory activities of HDPs and IDR peptides, these skin infection models are helpful tools when used complementarily with conventional microtiter plate assays that help bridge the gap between HDP discovery and potential drug development. Indeed, it could be argued that given the immunomodulatory potential of HDPs/IDR peptides, models involving both host and microbes provide superior potential to demonstrate efficacy under *in vivo*-like circumstances. Haisma et al. studied a group of synthetic peptides derived from P60.4Ac, a peptide based on LL-37, using a full-thickness skin model that was thermally wounded and infected with different strains of *S. aureus* (Haisma et al., 2014). One P60.4Ac derivative, Peptide 10, was found to reduce MRSA LUH14616 and mupirocin-resistant MRSA LUH15051 burden without affecting the level of IL-8 produced by thermally wounded skin (Haisma et al., 2014). A human epidermal model was later used to test the efficacy of various P60.4Ac formulations (Haisma et al., 2016). In particular, P60.4Ac could be stably formulated in a hypromellose gel, which was highly effective (from as low as 0.1% wt/wt) in eradicating planktonic and biofilm-associated MRSA on epidermal skin models without causing cytotoxicity (up to 2%) (Haisma et al., 2016). De Breij et al. used an *ex vivo* wounded skin infection model to confirm the activity of a hypromellose gel-formulated peptide SAAP-148 that showed superior efficacy in biofilm inhibition and eradication assays *in vitro* (de Breij et al., 2018). Likewise, a single treatment with SAAP-148 ointment eradicated acute infections and pre-formed biofilms of MRSA LUH14616 and *Acinetobacter baumannii* RUH875 from the wounded human skin (Figure 3) and abraded murine skin (de Breij et al., 2018). Ventress et al. showed that synthetic peptides derived from the EC2 domain of the tetraspanin CD9 could block the adherence of *S. aureus* to wounded skin models (Ventress et al., 2016). A role for LL-37 in the pathogenesis of the skin inflammatory condition rosacea could be demonstrated by the application of the anti-parasitic ivermectin into keratinocytes, reconstructed human epidermis and *ex vivo* skin models. This led to an anti-inflammatory effect mediated by suppressing expression of LL-37 and the serine protease kallikrein-5 (Thibaut de Menonville et al., 2017).

### Lung Models

The production of HDPs can be assessed in both lung and skin ALI models (Hertz et al., 2003; Beisswenger et al., 2006; Diamond et al., 2010). The comparison of HDP expressions between patients and healthy lung ALI cultures permit an understanding of the important roles of HDPs in respiratory diseases and can potentially identify key mechanisms or drug targets (Diamond et al., 2010; Zuyderduyn et al., 2011; Amatngalim et al., 2017). For example, Amatngalim et al. showed that the expression of non-typeable *Haemophilus influenzae* (NTHi)-induced human β-defensin 2 (*DEFB4*) and *S110A7*, but not LL37 (*CAMP*) or β-defensin 1 and 3 (*hDB-1* and *hBD-3*), were reduced in ALI from COPD patients when compared to non-COPD cultures (Amatngalim et al., 2017). In addition, cigarette smoke attenuated NTHi-reduced gene expression of *DEFB4, LCN2, S100A7, and CCL20*, while increasing IL-8 and IL-6 gene expression (Amatngalim et al., 2017). This study utilized a lung ALI model to demonstrate that the imbalance of reduced expression of HDPs and increased expression of pro-inflammatory cytokines might have contributed to the susceptibility to microbial infections of smokers and COPD patients. It was suggested that exogenous application of HDPs might provide a potential route of therapy in COPD (Amatngalim et al., 2017). Harcourt et al. used an ALI Calu-3 model to show that the efficacy of LL-37 in reducing respiratory syncytial virus (RSV) infections differs when the peptide is given as a prophylactic or therapeutic (Harcourt et al., 2016). When used in a prophylactic (pretreatment) regimen, LL-37 significantly reduced the intracellular viral genome counts as well as the production of cytokines and chemokines mediated by RSV (Harcourt et al., 2016). An ALI mode derived from primary HBE cells was used to show that the frog skin-derived HDP Esc (1-21) and its synthetic derivative Esc (1-21)-1c were both able to preserve epithelial layer barrier integrity upon *P. aeruginosa* challenge (Chen C. et al., 2017). It was also demonstrated that Esc (1-21)-1c, created by substituting two amino acids and using the corresponding D-enantiomer of Esc (1-21), had significantly reduced peptide-induced cellular toxicity and enhanced biostability and antibiofilm activity (Cappiello et al., 2016). In order to predict acute local lung toxicity, Ritter et al. utilized an ALI model with an optimized aerosol delivery system to evaluate different combinations of HDPs and nanocarriers (Ritter et al., 2020). This study demonstrated that the ALI culture system is more sensitive when compared to the submerged culture systems. It was suggested that the immediate contact between the deposited aerosol and epithelial surfaces contributed to a larger, faster bioavailability and more realistic estimation of the lowest observable adverse effect levels (Ritter et al., 2020).

### Intestinal Models

**Figure 4:**
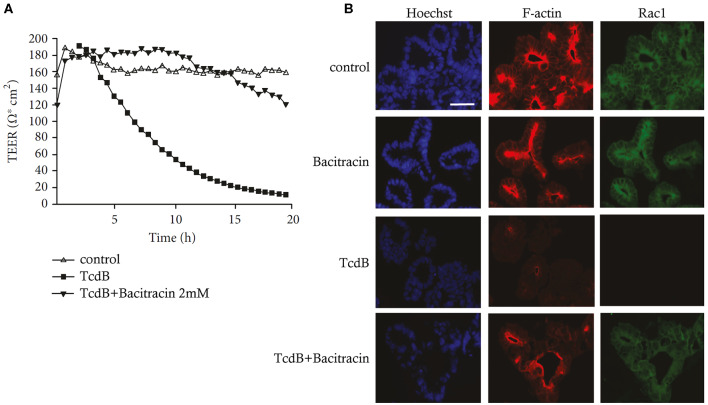
The antibiotic Bacitracin protected human gut epithelia and human intestinal organoids from *Clostridium difficile* Toxin TcdB. **(A)** Bacitracin preserves epithelial integrity of CaCo-2 monolayers from TcdB activity. Epithelial CaCo-2 monolayers grown on filters in a Transwell chamber were preincubated with or without Bacitracin (2 mM) for 30 min at 37°C and then stimulated apically with *Clostridium difficile* toxin B (TcdB, 6 ng/ml). The epithelial integrity was recorded by measuring the transepithelial electrical resistance (TEER) every 35 min over a time course of 20 h. **(B)** Bacitracin reduces the TcdB-induced glucosylation of Rac1 and destruction of F-actin in stem cell-derived human intestinal organoids. Intestinal organoids preincubated with or without Bacitracin (1 mM) for 30 min at 37°C were challenged with 60 ng/ml TcdB for 3 h. Nuclei, F-actin, and non-glucosylated Rac1 were specifically stained and visualized by confocal fluorescence microscopy. Bar = 50 μm. These results were from Zhu et al. (2019), published under the terms of the CC by 4.0 license. Full terms at https://creativecommons.org/licenses/by/4.0/. This figure combined Figures 3, 4 from the original article with permission from the corresponding author.

Other than growing as spheroids, both enteroid and colonoid cells can also be grown as polarized epithelial monolayers on permeable tissue culture membranes, allowing easy access to the lumen for infectious studies and drug screening assays (In et al., 2019). A recent study demonstrated the use of enteroid monolayers to perform screening of 2,000 candidate drugs, which shows that organoid models can provide a high-throughput drug screening tool (Kozuka et al., 2017). In other examples, transgenic expression of α-defensins in enteroids led to inhibition of *Salmonella* growth, illustrating the anti-bacterial activities of α-defensins at physiologically relevant conditions (Wilson et al., 2015). Furthermore single-cell RNA-Seq was used to demonstrate that Paneth-cell enriched enteroids mimic their *in vivo* counterparts, and can express a set of antimicrobial peptide marker genes including *Defa22, Defa21*, and *Ang4* (Mead et al., 2018). Intestinal organoids have also provided a model for the mechanistic characterization of the mode of action of bacitracin beyond its ability to inhibit cell wall synthesis in Gram-positive bacteria. Specifically, bacitracin can neutralize the *Clostridium difficile* TcdB toxin by preventing TcdB translocation into the cytosol of intestinal epithelial cells. Therefore reducing TcdB-induced glucosylation of Rac1 and destruction of F-Actin, which ultimately leads to the maintenance of epithelial integrity (Figure 4) (Zhu et al., 2019).

In addition, epithelial monolayers in a transwell plate provide researchers with the opportunity to co-culture the organoid with other immune cells (Hensel et al., 2014). Dekkers et al. generated intestinal organoids from rectal biopsies of cystic fibrosis (CF) patients and used a microscopic assay to functionally assess CFTR in these organoids (Dekkers et al., 2013). By using forskolin to raise the intracellular cyclic AMP levels and activate CFTR, ion uptake followed by fluid secretion into the lumen of the intestinal organoids was shown to lead to swelling of these organoids. However, intestinal organoids from CF patients showed reduced swelling compared to organoids from healthy individuals as a consequence of defective CFTR (Dekkers et al., 2013). Using these CF intestinal organoids and the forskolin assay, it was subsequently shown that the CFTR-modulating drug VX-770 (ivacaftor/kalydeco) can treat an uncharacterized rare *CFTR* genotype and improve clinical outcome for these patients (Dekkers et al., 2016). High-throughput screens have also been developed to identify drug sensitivities and putative protein kinase inhibitor using tumor organoids (Aboulkheyr Es et al., 2018; Phan et al., 2019).

## Limitations in Using Organoids

Although the various organoid culture systems have several advantages over conventional submerged *in vitro* models and *in vivo* animal models, there are certain hurdles that need to be overcome. First, most current organoid models consists of only the epithelial layer, which lacks the interactions with vascular, immune, and nervous systems (Xu et al., 2018). Researchers are addressing this limitation with continuous development and improvements of organoid models. For example, a multi-organ-on-chips system is currently being developed and refined, whereby multiple organ-on-a-chip systems can be connected, allowing real-time monitoring of the pharmacokinetics and pharmacodynamics of multi-organ interactions (Zhao et al., 2019). Co-culture systems are also utilized to increase the complexity and functionality of organoid models. For example, Workman et al. used a tissue-engineering approach to co-culture human iPSC-derived neural crest cells and human intestinal organoids (Heinz et al., 2003), giving rise to functional enteric nervous system -containing intestinal organoids that were capable of responding to calcium stimulation, demonstrating neuronal activities and expressing neurochemical markers (Workman et al., 2017). Using RNA-Seq analysis, the authors revealed the effects of the enteric nervous system on HIO development, revealing differentially expressed genes associated with digestive-tract development (e.g., EGF signaling components and TGF-β signaling factors), and absorptive (e.g., FABP2, LCT, TREH, SI, and MGAM) and secretory (MUC2, Paneth cell markers, and WNT3), lineage markers when compared to HIO alone (Workman et al., 2017). Moreover, this co-culture system could be used to mechanistically study of Hirschsprung's disease, caused by a mutation in the PHOX2B gene in human (Workman et al., 2017). Another study used a co-culture system containing primary human macrophages with enteroids, and showed that macrophages enhanced the barrier functions and maturity of enteroids (Noel et al., 2017).

Second, most organoid models currently reflect only the fetal stages of organ development. However, recent studies showed that transplantation into immune-compromized hosts promotes organoid maturation and enhances its functionality. For example, Dye et al. showed that transplantation of ESC-derived lung organoids into immune deficient NSG mice enhanced epithelial organization, structure development and cellular differentiation (Dye et al., 2016). In addition, the structure and cellular diversities of the transplanted lung organoids resemble adult human airways to a greater extent than do *in vitro* grown lung organoids (Dye et al., 2016). Third, most organoid models are relatively expensive due to the requirement for reagents and growth factors/inhibitors, and their dependence on extracellular matrix (such as Matrigel) and long-term culture (Xu et al., 2018). Animal-derived extracellular matrices, such as Matrigel, are prone to batch-to-batch variability and have poorly defined protein and growth factor compositions. The use of various growth factors and inhibitors during the organoid formation has the potential to mask drug responses. In addition, the process of organoid production is time consuming and labor intensive. To address some of these limitations, bioengineers are making an effort to create a well-defined culture media and extracellular matrix systems, such as collagen I gels and fibrin hydrogels, and utilize bioprinting technologies (Sachs et al., 2017; Yui et al., 2018). Lastly, current organoid models do not accurately recapitulate the metabolism of their parental organs (Fan et al., 2018). Further efforts in the development and refinement of organoid model building will help to overcome some of these limitations.

## Conclusion

Over the past few years, researchers had made extensive efforts to understand and improve the use of induced pluripotent stem cell-derived immune cells and organoid systems in the field of drug discovery (Figure 1). As discussed in some of the examples above, iPSC-derived epithelial, immune cell, and organoid models can be used as a powerful screening tool for precision therapy (Table 1). ALI cultures and organoids derived from primary cells or iPSC maintain the patient's genetic, functional and phenotypic signatures *in vitro*, allowing for accurate prediction of drug responses of individual patients (Gras et al., 2012; Bartfeld and Clevers, 2017; Oost et al., 2018; Kim et al., 2019; Leibel et al., 2019). In combination with genetic modification tools, such as lentiviruses or CRISPR/Cas9, organoids, and ALI cultures can be used to study the effects of specific genetic mutations in various diseases and identify potential drug targets and cell therapies (Schwank et al., 2013; Zhou et al., 2019). In addition, ALI and organoids can be expanded from small amounts of starting materials, and can be maintained over a long period of time to provide enough samples for a wide variety of laboratory analysis, such as efficacy, toxicology, multi-*omics* analyses (such as RNA-Seq, proteomic, and metabolomic). With regards to biofilm infections, which represent 65% of all human infections, are very resistant to antibiotics, and lack targeted therapies. ALI cultures and organoids offer a screening model for agents, including antimicrobial peptides to address such infections. We propose that future HDP and IDR peptide screens can be performed using both iPSC-derived and patient-specific ALI cultures/organoids with integrating multi-omics analysis, to not only identify new HDPs and IDR peptides, but also potentially determine which patients will benefit from a combination treatment using HDPs and antibiotics. Furthermore, ALI cultures and organoids can be used for toxicology testing to complement or replace animal testing.

## Author Contributions

K-YC, BW, AL, and BB wrote the manuscript. RH extensively edited the manuscript. All the authors reviewed and edited the final draft of the manuscript.

## Conflict of Interest

The authors declare that the research was conducted in the absence of any commercial or financial relationships that could be construed as a potential conflict of interest.
